# Cell cytoskeleton and proliferation study for the RANKL‐induced RAW264.7 differentiation

**DOI:** 10.1111/jcmm.16390

**Published:** 2021-03-19

**Authors:** Lingbo Kong, Rui Ma, Yang Cao, Wanli Smith, Yuan Liu, Xiaobin Yang, Liang Yan

**Affiliations:** ^1^ Department of Spine Surgery School of Medicine Honghui Hospital Xi'an Jiaotong University Xi'an China; ^2^ Department of Anesthesiology Xi'an Children Hospital Xi'an China; ^3^ Department of Psychiatry and Behavioral Sciences Johns Hopkins University Baltimore MD USA

**Keywords:** osteoclast, protocol, RANKL, RAW264.7CRL‐2278, RAW264.7TIB‐71

## Abstract

Although document studies (including ours) have been reported the achieved in vitro osteoclastic cellular model establishment from the RAW264.7 cell lineage, there was no study directly reported that American Type Culture Collection (ATCC) cell bank has various RAW264.7 cell lineages. Besides that, for our knowledge there was only one study compared the two different RAW264.7*^TIB‐71^* and RAW264.7*^CRL‐2278^* cell lineages for their osteoclastic differentiation, and they concluded that the RAW264.7*^CRL‐2278^* demonstrated to generate much osteoclast than RAW264.7*^TIB‐71^*. However, on the contrary to their results we noticed the fusion of RAW264.7*^TIB‐71^* in our previous studies was much compromising. Therefore, we try to explore the two cell lineages for their properties in osteoclastic differentiation with an in‐depth cellular cytoskeletal study. Our current study has showed that comparing to the RAW264.7*^CRL‐2278^*, RAW264.7*^TIB‐71^* demonstrated a much higher efficacies for RANKL‐stimulated osteoclastic differentiation. Besides that, in our depth cytoskeletal studies, we found that the RANKL‐induced RAW264.7*^TIB‐71^* cells could finally differentiate into mature osteoclasts. However, regardless the various pre‐treatment conditions, there was no mature osteoclast formed in RANKL‐induced RAW264.7*^CRL‐2278^* cell lineage.

## 1 INTRODUCTION

Although document studies (including ours) have been reported the achieved in vitro osteoclast model establishment from the RAW264.7 cell lineage, there were no reports about various RAW264.7 cell lineages existing in American Type Culture Collection (ATCC) cell bank, such as ATCC^®^TIB‐71, ATCC^®^CRL‐2278, ATCC^®^SC‐6003, ATCC^®^SC‐6004 and ATCC^®^SC‐6005 (property details in Table 1), in which each cell lineage possesses significant different properties that might lead a quite different study result.

**TABLE 1:** ATCC RAW264.7 cell lineage list and properties (Information from ATCC)

Cell lineage	ATCC CAT number	Cell type	Properties
RAW264.7	ATCC^®^ TIB‐71™	Macrophage	This line was established from a tumour induced by Abelson murine leukaemia virus
RAW 264.7 (gamma NO^(−)^)	ATCC^®^ CRL‐2278™	Monocyte, Macrophage	Unlike the parental line, RAW 264.7 gamma NO(−) does not produce nitric oxide upon treatment with interferon gamma alone, but requires LPS for full activation (the iNOS promoter linked to a luciferase reporter gene is also unresponsive to IFN‐alone).
			This property makes its behaviour more like that of normal macrophages from some commonly used strains of mice.
RAW 264.7 (LRRK2 parental)	ATCC^®^ SC‐6003™	Macrophage	This cell line can be used as a control alongside LRRK2 KO RAW 264.7 (ATCC^®^ SC‐6004™) or LRRK2 T1348N mut RAW 264.7 (ATCC^®^ SC‐6005™) in experiments determining the role of LRRK2 in Parkinson's disease
RAW 264.7 (LRRK2 KO)	ATCC^®^ SC‐6004™	Monocyte/macrophage	This cell line can be used alongside its wild‐type control LRRK2 parental RAW 264.7 (ATCC^®^ SC‐6003™) to investigate Parkinson's disease.
RAW 264.7 (LRRK2 T1348N mut)	ATCC^®^ SC‐6005™	Monocyte/macrophage	This cell line can be used alongside its wild‐type control LRRK2 parental RAW 264.7 (ATCC^®^ SC‐6003™) to investigate Parkinson's disease.

Besides that, for our knowledge there was only one study conducted by Nicolin et al^1^ compared the two different RAW264.7 ATCC^®^TIB‐71 (RAW264.7*^TIB‐71^*) and RAW264.7 ATCC^®^CRL‐2278 (RAW264.7*^CRL‐2278^*) lineages for their osteoclastic differentiation, and they concluded that RAW264.7*^CRL‐2278^* demonstrated to generate much osteoclast than RAW264.7*^TIB‐71^*. However, on the contrary to their results we noticed the fusion of RAW264.7*^TIB‐71^* in our previous studies was much compromising; therefore, we try to explore the two cell lineages for their properties in osteoclastic differentiation with an in‐depth cellular cytoskeletal study.

## 2 MATERIALS AND METHODS

### 2.1 Cell cultures and treatments

RAW264.7*^TIB‐71^* and RAW264.7*^CRL‐2278^* were purchased from ATCC. Cell properties are detailed in Table 1. The culturing and induction agents are detailed in Table 2. In our current study, RAW264.7*^TIB‐71^* cells were cultured as our previous studies reported.^2^, ^3^ Briefly, RAW264.7*^TIB‐71^* was seeded into 96‐well plate by the density of 2 × 10^3^ cells/mL in DMEM, containing L‐glutamine (4 mM), FBS (10%), sodium bicarbonate (1.5 g/L) and glucose (4.5 g/L). Besides that, RAW264.7*^CRL‐2278^* was initially seeded into the 96‐well plate at the same density to RAW264.7*^TIB‐71^* (2 × 10^3^ cells/mL) in RPMI‐1640 (according to ATCC's instruction), which contains L‐glutamine (2 mM), FBS (10%), sodium bicarbonate (1.5 g/L) and glucose (4.5 g/L), HEPES (10 mM) and sodium pyruvate (1 mM) before the osteoclastic induction.

**TABLE 2:** Cell culture and induction agent information

Reagent	CAT number	LOT number	Company
IFN‐γ	315‐05	061798	PeproTech
RPMI‐1640	30‐2001	81228190	ATCC
Foetal bovine serum	100‐106	A39E00F	Gemini Bio‐Product
MEM alpha modification	SH30265.01	AB214569	HyClone
RANKL	315‐11C‐10UG	0715612F1917	PeproTech
Lipopolysaccharides	L2880‐10MG	297‐473‐0	Sigma‐Aldrich
TRITC Phalloidin	40734ES75	T2811131	Yeasen
Mito Tracker Green FM	40742ES50	M7924980	Yeasen
DMEM	SH30022.01	AD17218273	HyClone
M‐CSF	SRP3221‐10UG	387‐09901	Sigma‐Aldrich

### 2.2 Osteoclastic grouping and induction

For osteoclastic differentiation, we induced the RAW264.7*^TIB‐71^* as we previously reported.^2^ Besides that, we modified the induction method for RAW264.7*^CRL‐2278^* in our current study in order to abrogate the culturing divergences with RAW264.7*^TIB‐71^*. Specifically, we changed the induction from RPMI‐1640 medium into α‐MEM at the first induction day. Both cell lineages were inducted for 7 days in several culture group with different stimulate conditions, the grouping details are listed in Supplementary Data, and the concentrations of each stimulator were determined after our preliminary studies.

### 2.3 Morphological study and TRAP staining

After initially seeding, both cell lineages were allowed to grow and attach the wall for 24 hours. After 24 hours, we starting to treat cells with cocktailed stimulators for 10 days, and we counted this initial treating day as day 0. Subsequently, cellular morphological alterations were recorded and evaluated at days 1, 2, 3, 4, 5, 6 and 7 by an inverted microscope Nikon (Nikon) and NIS‐Element analysis software (Nikon) for image analysis. TRAP staining kits were purchased from Sigma‐Aldrich Co. According to instructions, after PBS washing two cell lineages were fixed by paraformaldehyde (4%) lasting for 30 minutes. Subsequently, both cell lineages were washed by PBS and then stained by TRAP solution at 37°C in 1 hour, the staining results were evaluated by Nikon (Nikon) and NIS‐Element analysis software (Nikon) for image analysis.

### 2.4 Cytoskeleton F‐actin staining and Scanning electron microscopy

We performed cytoskeletal fibrous actin (F‐actin) staining for two subjects in order to observe the podosome patterns and sealing zone formation in each cell linages. Briefly, two cell lineages were washed by PBS, after washing cells were incubated in formaldehyde (4%) for 15 minutes fixing, then cells washed by PBS for three times. In order to increase permeability, before the phalloidin red incubation, the Triton X‐100 (0.1%) was added for 1 minutes. After 1 hour of incubation, DAPI was used for nucleus staining. F‐actin–stained cytoskeletons were visualized and quantified with a fluorescence microscope Nikon (Nikon) and NIS‐Element analysis software (Nikon) for image analysis. Besides that, we performed SEM. Briefly, matrices were then fixed by sodium cacodylate (0.1 M) containing glutaraldehyde (2.5% (v/v)); the final cells were recorded by the JSM6700F electron microscope.

### 2.5 Statistical analysis

Final data results were analysed by ‘two‐way ANOVA’ and ‘Student's *t* test’ methods by the software GraphPad Prism (version 8). Final, the value of *P* < .05 was defined as statistical significant in our current study, the data presented by the mean ± standard deviation (SD) manner.

## 3 RESULTS

### 3.1 The multinucleated cells from RAW264.7*^TIB‐71^* and RAW264.7*^CRL‐2278^* showed a significant difference during osteoclastic induction

**FIGURE 1:**
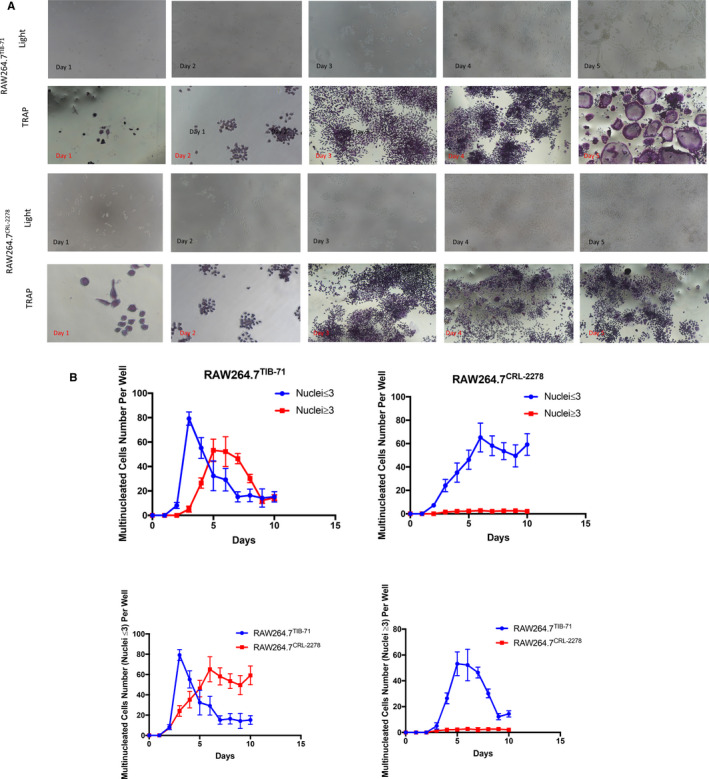
Efficacy of RANKL induced TRAP multinucleated cell formation from RAW264.7*^TIB‐71^* and RAW264.7*^CRL‐2278^*: (A): Light view and TRAP staining results (B):Time points for multinucleated cell formation

Previous, Nicolin et al^1^ has reported that RAW264.7*^CRL‐2278^* showed a faster and stronger osteoclast differentiation than RAW264.7*^TIB‐71^* within a 7 days of induction. However, our previous study has showed that RAW264.7*^TIB‐71^* could be induced to gain osteoclasts in a short‐term induction (within 3 days from the initial induction).^2^ Besides that, we have noticed that in the study of Nicolin, authors presented a smaller osteoclast in their study for both cell lineages. However, in our previous studies,^2^, ^3^ we have showed that the RAW264.7*^TIB‐71^* could be induced into giant osteoclasts. Moreover, even there were vast studies used the osteoclasts derived from RAW264.7 (RAW‐OCs) for their in vitro osteoclastic studies, there were few reports noted the brand of cell lineages for each usage. Therefore, in our present study we performed a subtle compare for these two cell lineages (Figure 1A).

In our current study, we found RAW264.7*^TIB‐71^* could be introduced as osteoclast by RANKL independently stimulation in α‐MEM. Besides that, RANKL independently stimulation could also successfully induce osteoclastic differentiation in DMEM and 1640 media, but the α‐MEM showed a significantly high producing efficacy for osteoclastic differentiation (data not be presented). However, even there were numbers of fused cells during RAW264.7*^CRL‐2278^* culture, there were no multinucleated cells (nuclei ≥ 3) in the RANKL independently induced group and the LPS + INF‐γ–induced group. However, interestingly, we found that under the fully activated condition (LPS + INF‐γ), the RAW264.7*^CRL‐2278^* could be induced into multinucleated cells by RANKL independently treatment, but the numbers of nuclei are significantly less than RANKL independently induced RAW264.7*^TIB‐71^*. These results interestingly demonstrated that, even under the fully activated condition of RAW264.7*^CRL‐2278^*, this cell lineage possesses a completely different property comparing to its parental cell lineage RAW264.7*^TIB‐71^*. Interestingly, consist with our previous studies, in our present study, we noticed M‐CSF has not shown significantly enhancing effects on osteoclastic formation for both RAW264.7*^TIB‐71^*and RAW264.7*^CRL‐2278^* (Figure 1B).

For over 30 years, TRAP staining has been demonstrated as one of the most important and reliable methods for identifying osteoclasts and their precursors.^4^, ^5^, ^6^ Besides that, TRAP staining also demonstrated an effective manner for nucleus counting.^7^ Therefore, we performed TRAP staining for initial multinucleated cell counting. Consist with previous studies, both RAW264.7*^CRL‐2278^* and RAW264.7*^TIB‐71^* groups could generate TRAP‐positive multinucleated cells. However, the size and nucleus numbers of fused cells are significantly different for these two cell lineages. As shown in figures, from the induction of day 3, the numbers of multinucleated cells in the RANKL‐induced RAW264.7*^TIB‐71^* group have significantly increased than the RAW264.7*^CRL‐2278^* group. However, in the 3rd day of induction, these multinucleated cells demonstrated a smaller morphological cellular shape with presenting a less nuclei in both cell lineages. Interestingly, although the morphological and nucleus numbers have not shown a significant increase in RAW264.7*^CRL‐2278^* in the 5th day of induction, the size and nucleus numbers in the RANKL‐induced RAW264.7*^TIB‐71^* group are significantly increased (*P* < .05) (Figure 1).

### 3.2 Cytoskeletal structure F‐actin stain and SEM morphological study showed there has no sealing zone formed in RAW264.7*^CRL‐2278^*

**FIGURE 2:**
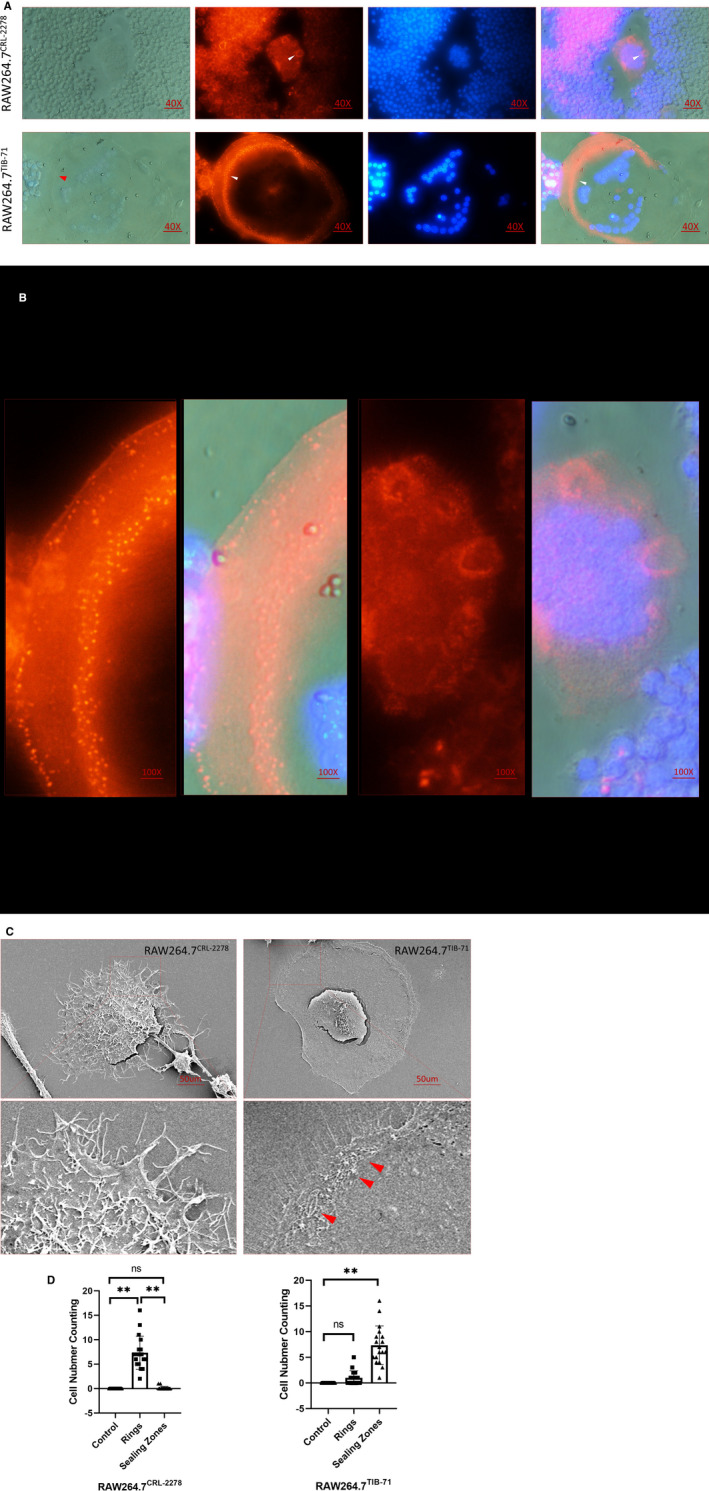
Cytoskeletal structure F‐actin stain and SEM morphological study for sealing zone formation: (A) F‐actin staining for sealing zone (arrows for ‘ring’ structure in up row figure; for ‘sealing zone’ for down row figure); (B) magnified figures of (A); (C) SEM view (arrows for ‘sealing zone’); (D) quantification results

Morphological changes characterize the degree of maturation and functional capabilities of osteoclasts. Specifically, at the late stage of osteoclast differentiation (osteoclastogenesis), matured osteoclast adhesion to the bone surface followed cytoskeletal organelles rearrangements. These rearrangements allowed polarization of osteoclasts to form four functional domains, including ruffled border with an isolated milieu of resorptive microenvironment and podosome patterned sealing zone (SZ). In that, SZ is one of the critical functional domains that segregates the resorptive microenvironment between osteoclast adhesion zone and bone tissue. Therefore, we performed F‐actin assay to assess the osteoclastic cytoskeletal SZ structure formation estimating osteoclastogenesis for RAW264.7*^CRL‐2278^* and RAW264.7*^TIB‐71^* in various inducing conditions. Our results showed that compared with RAW264.7*^CRL‐2278^* the numbers of F‐actin–rich SZ were significantly increased in RANKL‐induced RAW264.7*^TIB‐71^* induction group (Figure 2A‐B,D).

Besides the immunofluorescence for F‐actin observation, our current study also for the first time compared the morphological differences between RANKL‐induced RAW264.7*^CRL‐2278^* in fully activated condition with RANKL‐induced RAW264.7*^TIB‐71^* by SEM. Our results showed the morphological differences between the two cell lineages can be noted, RANKL induced fully activated RAW264.7*^CRL‐2278^* cells shaped in wedge shape with much microvilli in cellular dorsal surface, which in turn the RANKL induced RAW264.7*^TIB‐71^* cells presented an flat and smooth surface in cellular dorsal periphery. Moreover, the SZ could be clearly noted in RANKL‐induced RAW264.7*^TIB‐71^* cells (Figure 2C).

### 3.3 The pattern and number of podosome formed in RAW264.7*^TIB‐71^* and RAW264.7*^CRL‐2278^* are significantly different

**FIGURE 3:**
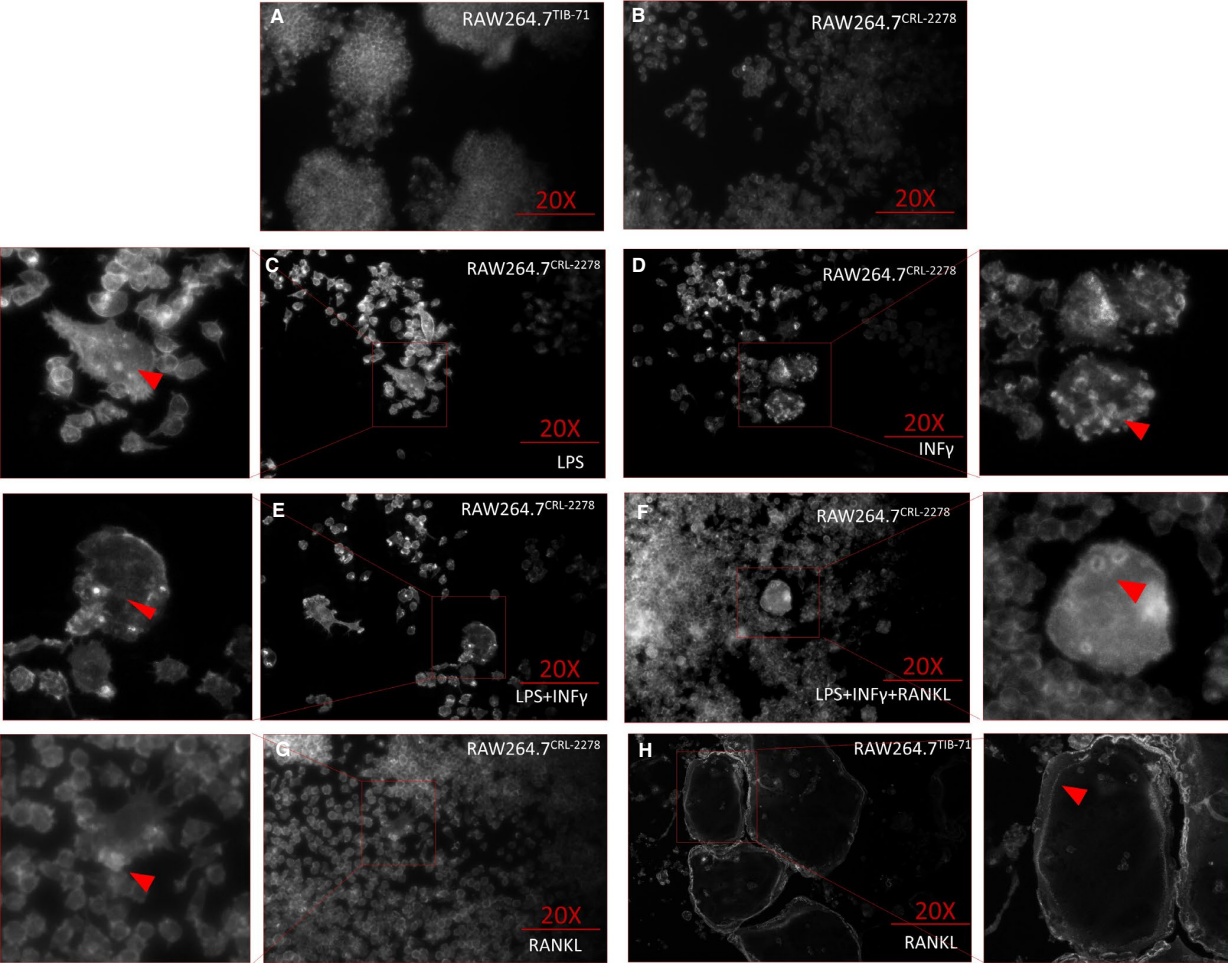
Podosome patterns in RANKL induced RAW264.7*^TIB‐71^* and RAW264.7*^CRL‐2278^*

In fact, the SZ is composed of tightly packed dot‐like attachment organelles called podosomes. Besides the podosome is one of the key organelles for osteoclast motility and bone resorption functions, it could able to organize into various patterns in the different stages during osteoclast formation. In general, podosomes could collectively pattern into ‘clusters’, ‘rings’ in immature osteoclasts and with the maturing process finally pattern into ‘belt’‐like SZ in matured osteoclasts. In our figure results presenting, the cluster pattern could be seen in figure, which present a ‘relaxed’ architecture. However, with the lasting induction podosome ‘clusters’ could evolve to transient ‘rings’. Interestingly, in our current study, the podosome patterned a significant number of SZs in RAW264.7*^TIB‐71^*; however, compared to the RAW264.7*^TIB‐71^* the podosome could only pattern ‘rings’ structure in RAW264.7*^CRL‐2278^*. As previously reported, SZ is associated with a functional activity and is at the periphery of the osteoclasts. Besides that, we could find these morphological details in both immunofluorescent and SEM figure results only for RAW264.7*^TIB‐71^* cell lineages. Moreover, consist with previous report, in the SEM scan results, we could observe podosomes presented an increasingly interconnectivity in the SZ (Figure 3).

## 4 DISCUSSION

Osteoclast formation and functional activity are important for bone homeostasis, which is key study target for bone biology.^8^ As we previously reported, RAW264.7 cell lineage not belongs to osteoclastic homogeneous.^9^ It is prevailing in the fields of bone homeostasis study because it merits advantages for providing a much easier accessible osteoclastic cellular model. However, during our usage, we found methodologies for RAW‐OCs still elusive and existing controversies. For instance, several studies reported that RAW‐OCs were generated by M‐CSF and RANKL co‐stimulation,however, we previously demonstrated the RAW‐OCs could be generated by RANKL or LPS independently treatment.^10^, ^11^, ^12^, ^13^ These existing discrepancies might lie in the unique properties of RAW264.7 cell lineages, such as keep changing in its phenotypes during passage process.^9^, ^14^, ^15^ Besides that, regardless the vast usages of RAW264.7 cells in various studies, few studies clearly involved the catalog numbers that could be a crucial but unnoticed issue cause the different results for using RAW264.7 cells in same culturing and inducing condition. Specifically, we notice there still lacks extensive study for the differences between RAW264.7*^CRL‐2278^* and RAW264.7*^TIB‐71^*. To our knowledge, there was only one study conducted for comparing these two cell lineages for their osteoclast formation ability.^1^ Their results demonstrated that the RAW264.7*^CRL‐2278^* showed a stronger ability in osteoclast formation. Interestingly, on the contrary, according to our own experiences, we found the RAW264.7*^TIB‐71^* could stably fused into bigger bone resorptive osteoclast with a greater number of nuclei (≥10). Therefore, in our current study we conducted an extensive study for these two cells, which provide more details and evidences for RAW‐OC culturing methodology.

Osteoclasts could commonly be characterized by two criteria: the multiple nuclei (≥3) and TRAP activity.^4^, ^12^, ^16^, ^17^ In that, TRAP activity is normally performed to evaluate activity of osteoclastogenesis. However, TRAP could also express in other hematopoietic‐originated cell lineages such as macrophages.^18^ Therefore, studies have to examine the osteoclast nucleus numbers and their associated osteoclastic activities, which demonstrated a significant correlation in the nucleus number and cellular size with the depth of the subsequent bone lacunae.^19^, ^20^, ^21^ Commonly, in bone physiological homeostasis, osteoclasts could contain 3 to 10 nuclei. However, in the bone pathological homeostasis, the numbers of nuclei and the size of osteoclast are commonly noticed, such as Paget's disease, periodontal disease and rheumatoid arthritis (RA).^22^, ^23^ Constant to these observations, document studies, including our in vitro assays, have shown that the larger osteoclasts which with more nuclei (ie ≥ 10 nuclei) are more likely to be in a resorptive activities than smaller counterpart with less nuclei (ie, ≤ 5 nuclei). That might lie in that the larger osteoclasts with more nuclei (≥10 nuclei) presented a significantly higher basal pHi than smaller counterpart, and that high pHi reflecting a considerable V‐ATPase activity in osteoclasts. In our current study, we found RAW264.7*^CRL‐2278^* failed to form OCs under several stimulating conditions; instead, RAW264.7*^CRL‐2278^* could generate multinucleated cells under the condition of RANKL‐stimulated LPS + INF‐γ group. However, on the contrary to previously reported, comparing the RANKL induced osteoclasts from RAW264.7*^TIB‐71^* osteoclasts from RANKL induced RAW264.7*^CRL‐2278^* presented a significantly small size and less nuclei numbers.

Image analysis has been a promising tool for quantitation of complex biological processes, and image analysis could enable assessment for the correlation between the osteoclastic activity and the cellular maturation state, which further stratified the treatment efficacies. Therefore, we further performed the immunofluorescence and SEM for investigating the cytoskeleton of these cells. In fact, podosome could be observed in a wide variety of cell types, including macrophages and dendritic cells. An actin‐rich organelle podosome plays critical role in various cellular functions, such as motility, adhesion and matrix invasion. During the osteoclast formation and maturation, podosomes initially are observed from SEM for their localized on ventral membrane of the osteoclast at a contact site against the underlying substrate. During the osteoclastic differentiation, podosomes rapidly pattern into ‘clusters’ and ‘rings’; further with the maturation of osteoclasts, podosomes finally pattern into the SZ. Most importantly, the highly organized SZ of the osteoclasts is a unique apparatus, which could be only observed in matured osteoclasts, and serves for both osteoclastic cellular adhesion and extracellular matrix resorption. Our current study results showed during the whole culturing, there was no SZ patterned in *RAW264.7^CRL‐2278^* cell lineage.

## CONFLICT OF INTEREST

The authors declare that they have no competing interests.

## AUTHOR CONTRIBUTIONS

**Lingbo Kong:** Conceptualization (equal); Data curation (equal); Formal analysis (equal); Funding acquisition (equal); Investigation (equal); Methodology (equal); Project administration (equal); Resources (equal); Writing‐original draft (equal). **Rui Ma:** Investigation (equal); Methodology (equal); Writing‐review & editing (equal). **Yang Cao:** Data curation (equal); Investigation (equal); Software (equal). **Xiaobin Yang:** Methodology (equal). **Liang Yan:** Conceptualization (equal); Data curation (equal); Resources (equal); Supervision (lead). **Yuan Liu:** Validation (equal); Writing‐review & editing (equal). **Wanli Smith:** Conceptualization (equal); Methodology (equal).

## ETHICS APPROVAL AND CONSENT TO PARTICIPATE

None animal studies have involved in the current study.

## CONSENT FOR PUBLICATION

The manuscript is approved by all authors for publication.

## Supporting information

Supplementary Data

## Data Availability

All data and materials were included in the manuscript available.
